# Selective or stepwise removal of deep caries in deciduous molars: study protocol for a randomized controlled trial

**DOI:** 10.1186/s13063-014-0525-9

**Published:** 2015-01-06

**Authors:** Falk Schwendicke, Hardy Schweigel, Marina Agathi Petrou, Ruth Santamaria, Werner Hopfenmüller, Christian Finke, Sebastian Paris

**Affiliations:** Department of Operative and Preventive Dentistry, Charité - Universitätsmedizin, Aßmannshauser Str 4-6, Berlin, 14197 Germany; DMG Dental Material Gesellschaft, Department of Clinical Research, Elbgaustr 248, Hamburg, 22547 Germany; Department of Operative Dentistry, Periodontology and Preventive Dentistry, RWTH University of Aachen, Pauwelsstr 30, Aachen, 52074 Germany; Department of Preventive and Paediatric Dentistry, Ernst-Moritz-Arndt University of Greifswald, Rotgerberstr 8, Greifswald, 17487 Germany; Institute of Medical Biometrics and Clinical Epidemiology, Charité - Universitätsmedizin, Hindenburgdamm 30, Berlin, 12203 Germany; Department of Orthodontics, Dentofacial Orthopedics and Pedodontics, Charité - Universitätsmedizin, Aßmannshauser Str 4-6, Berlin, 14197 Germany

**Keywords:** Caries, Costs, Dentin, One-step excavation, Partial excavation, Two-step excavation

## Abstract

**Background:**

For treating deep caries lesions, selective or stepwise (one- and two-step) incomplete excavation seems advantageous compared with complete caries removal. However, current evidence regarding the success, as defined by not requiring any retreatments, or survival of teeth after different excavations is insufficient for definitive recommendation, especially when treating deciduous teeth. Moreover, restoration integrity has not been comparatively analyzed longitudinally, and neither patients’, dentists’ or parents’ preferences nor the clinical long-term costs emanating from both initial and retreatments have been reported yet.

**Methods/Design:**

The planned study is a prospective multicenter, two-arm parallel group, randomized controlled clinical trial comparing selective and stepwise excavation in deciduous molars with deep, active caries lesions without pulpal symptoms. We will recruit 300 children aged between three and nine-years-old with a minimum of one such molar. Patients participating in another study, or those with systemic diseases, disabilities or known allergies to used materials as well patients with teeth expected to exfoliate within the next 18 months will be excluded. After inclusion, sequence generation will be performed. Initial treatment will follow dental routine. During excavation, leathery, moist and reasonably soft dentin will be left in proximity to the pulp followed by adhesive restoration of the cavity. Afterwards, patients’, dentists’ and parents’ subjective assessment of the treatment will be recorded using visual analogue or Likert scales. Re-examination will be performed after six months, and only then teeth will be allocated to one of the two interventions. Selectively excavated teeth will not be treated further, whilst for stepwise caries removal, a second excavation will be performed until only hard dentin remains. Clinical re-evaluations will be performed after 12, 24 and 36 months. Restorations will be reassessed using modified Ryge criteria. Objectively or subjectively required retreatments will determine success and survival. Retreatments will be evaluated both subjectively and regarding generated costs.

**Discussion:**

Based on the results of the trial, decision-making for treating deep caries lesions in deciduous molars based on multiple criteria should be feasible.

**Trial registration:**

Clinicaltrials.gov identifier: NCT02232828 (registered on 29 November 2014).

## Background

The treatment of deep caries lesions is associated with significant risks for the pulp, including pulpal exposure and postoperative pulpal complications, which might eventually compromise the retention of the tooth [1]. Moreover, treating deep lesions might be associated with pain and subjective burden both during and after treatment, and might generate long-term costs due to retreatments being required [2,3].

For deciduous teeth, various treatments for deep lesions have been described: complete excavation aims at removing all infected and affected carious dentin, with the inherent risk of pulpal exposure. In contrast, stepwise (two-step) excavation leaves carious dentin after the initial excavation step, then seals residual caries under a temporary restoration, and re-enters the cavity in a second step to eventually attempt complete excavation. This approach is thought to facilitate arrest and remineralization of the lesion and to induce development of tertiary dentin, thereby reducing the risk of pulpal exposure and postoperative complications after the second excavation step [4,5]. Since several studies found sealed residual lesions to be clinically and microbiologically arrested, the need to re-enter was increasingly questioned within the last decade [6]. Selective (one-step) incomplete or partial excavation seals carious dentin under a definitive restoration, omitting any re-entry [7]. Sealing the lesion is thought to deprive residual bacteria from dietary carbohydrates and has been found to exert significant antibacterial effects, thus arresting the lesion [8,9].

However, doubts remain regarding the effects of sealed carious dentin on the long-term quality of the restoration [10]. Moreover, it remains unknown if patients prefer one treatment over the other, which might be especially relevant when treating children. Several studies comparing complete with selective or stepwise excavation of deciduous teeth have been published, but only one three-arm study compared selective with stepwise excavation of primary teeth (Table 1). In addition, none of these studies assessed patient- or dentist-centered outcomes, that is, preferences, or analyzed clinically assessed long-term costs emanating from both excavations.

**Table 1:** **Published randomized controlled trials comparing different excavations of dentin caries in deciduous molars**

**Study**	**Study type; setting; country**	**Participants (age); teeth; lesion**	**Intervention (number of teeth)**	**Control (number of teeth)**	**Follow-up, drop-out**	**Pulp exposure (PE)**
						**Pulp symptoms (PS)**
						**Failure (F)**
						**Caries progression (CP)**
Leksell *et al.* 1996 [11]	Multicenter parallel-group RCT; university and clinics; Sweden	116 (6-16); 134 primary molars; caries with ‘risk of pulp exposure’	Stepwise (64) (‘remaining innermost layer of carious dentin’ left); re-entry after 8-24 weeks	CR (70) (‘hard’)	24 weeks, 4.3% yearly	PE: 18% stepwise, 40% CR
						PS: Non-exposed teeth remained asymptomatic
						F: 0% stepwise, 0% CR
Foley *et al*. 2004 [12]	Split-mouth RCT; university; Scotland	44 (3-9); 120 primary molars; dentin caries	Selective (36) restored with BCC and GIC; one-step (43) restored with just GIC	CR (41)	24 months, 11% yearly	PS: More abscesses in BCC group
						F: 23% GIC, 33% BCC, 22% CR
						CP: Caries increase highest in CR
Ribeiro *et al*. 1999 [13]	Parallel-group RCT; university; Brazil	38 (7-11); 48 primary molars; dentin caries without risk of pulp exposure	Selective (24) (‘partially removed, soft and moist dentine left’)	CR (24) (dye)	12 months, 0% yearly	PS: 0% selective, 4% CR
						F: 0% selective, 4% CR
						CP: 25% selective
Orhan *et al*. 2010 [14]	Parallel-group RCT; university; Turkey	123 (4-15); 154 (94 deciduous and 60 permanent) molars; caries >3/4 dentin	Stepwise (50) (‘thin layer of soft tissue left’); Stepwise (49); re-entry after 3 months	CR (55)	12 months, 0% yearly	PE: 6% selective, 8% stepwise, 22% CR
						PS: 0% selective, 2% stepwise, 5% CR
						F: 0% selective, 2% stepwise, 5% CR
Magnusson and Sundell, 1977 [15]	Parallel-group quasi-RCT; university; Sweden	62 (5-10); 110 primary molars; caries ‘considered for stepwise excavation’	Stepwise (55) (‘soft layer of dentin over the pulp’); re-entry after 4-6 weeks	CR (55) (‘until hard’)	No follow-up	PE: 11% stepwise, 53% CR
						PS: 5% stepwise, CR not followed-up
Heinrich *et al*. 1991 [16]	Parallel-group RCT; university; Germany	125 (6-7); 125 primary molars; deep caries	Stepwise (52) (‘slightly soft’); re-entry after 6-8 weeks	CR (52) (‘hard with explorer’)	16 months, 12% yearly	PE: 15% stepwise, 31% CR
						PS: 6% stepwise, 13% CR
						F: 6% stepwise, 13% CR
Lula *et al*. 2009 [17]	Parallel-group RCT; university; Brazil	30 (5-8); 36 primary molars; caries >1/2 dentin	Selective (18) (‘only superficial necrotic dentin removed from the pulpal and axial walls’)	CR (18) (dye)	6 months, 5.7% yearly	PE: 0% selective, 22% CR
						PS: 0% selective, 7% CR
						F: 0% selective, 14% CR
Phonghanyudh *et al*. 2012 [18]	Bi-centered parallel-group RCT; dental hospitals; Thailand	276 (6-11); 276 primary molars; caries ≥1/3	Selective (92) (‘soft carious tissues at EDJ completely removed, without further removal of carious dentin’)	CR (92)	12 months, 2.5% yearly	PE: 0% selective, 2% CR
						PS: 1% selective, 2% CR
						F: 18% selective, 14% CR restoration failure
						CP: No caries progression in any group
Franzon *et al*. 2014 [19]	Split-mouth RCT; university; Brazil	51 (3-8); 124 primary molars; Caries in ‘inner quarter of dentin’	Selective (61) (‘leathery’)	CR (67)	24 months, 2.0% yearly	PE: 2% selective, 28% CR
						PS: 10% selective, 0% CR

BCC: black copper cement, CR: complete caries removal, EDJ: enamel-dentinal junction, GIC: glass ionomer cement, RCT: randomized controlled trial.

### Objectives and hypotheses

The study aims at comparing the success (the probability of not requiring any re-interventions) and the survival (the probability of not requiring tooth removal) of selectively excavated versus stepwise excavated vital, non-symptomatic deciduous molars with deep lesions. In addition, we assess the restoration integrity of selectively excavated versus stepwise excavated deciduous molars, evaluate the preference of patients, parents and dentists for one of both strategies, and comparatively assess the costs associated with each strategy.

Our primary hypothesis is that success rates differ significantly between selectively excavated and stepwise excavated teeth. Secondary hypotheses are that restoration integrity is assumed to significantly differ between selectively excavated and stepwise excavated teeth. Moreover, we hypothesize that patients’, parents’ and dentists’ preference is significantly different for selectively excavated versus stepwise excavated teeth. Eventually, both initial and long-term costs of excavation methods are supposed to significantly differ.

## Methods/Design

### Overview

The planned study is a secondary care-based prospective, multicenter, two-arm parallel-group, randomized controlled trial at three pediatric university dental clinics in Germany: the Charité - Universitätsmedizin Berlin, the Ernst-Moritz-Arndt University Greifswald and the RWTH University of Aachen. We plan to enroll 300 patients with one or more deeply carious, sensitive and non-symptomatic deciduous molar. One molar per patient will be randomly allocated to receive one of two treatments (selective or stepwise excavation). The total follow-up time will be three years after completion of the initial treatment. Success, survival and restoration integrity will be assessed after one, two and three years. Patients’, parents’ and dentists’ preference will be assessed after each treatment using visual analogue or Likert rating scales. Costs will be assessed for the initial and follow-up treatments and will be based on a micro-costing approach. The study has been approved by the ethics committee of the Charité - Universitätsmedizin Berlin (approval number: EA4/057/14), the Ernst-Moritz-Arndt University Greifswald (approval number: BB 112/14) and the RWTH University of Aachen (approval number: EK 283/14). The study is registered at Clinicaltrials.gov (identifier: NCT02232828).

### Setting and participants

The study will take place in three dental university clinics in Eastern, Northern and Western Germany. All are publically funded teaching hospitals. We will include children aged between three and nine-years-old with a minimum of one sensitive, clinically and radiographically non-symptomatic, retainable, deeply carious deciduous molar with a caries lesion involving either only the occlusal or the occlusal and exactly one proximal (mesial or distal) surface. The lesion will be required to radiographically extend into the inner third of the dentin (D3) and show signs of activity, such as plaque retention, papillary bleeding and/or softness of the surface [20]. Patients will require parental consent for participation. In addition, patients’ cooperation for treatment under no or only local anesthesia will be expected.

Patients participating in another study, patients with parents planning to move away within the next three years, patients who are not residents of the federal states of Berlin, Brandenburg, Mecklenburg-Vorpommern and Nordrhein-Westfalen, as well as patients with systemic diseases or disabilities will not be included. Patients with known allergies to dental materials used within the study as well as those with teeth expected to exfoliate within the next 18 months will also not be included.

### Sample size

The required sample size was calculated based on the primary outcome parameter, success (see below). We anticipate a hazard ratio of 1.3 [21] of stepwise excavated compared with selectively excavated teeth. If α = 0.05 and 1-β = 0.9, a sample size of n = 66 per group is required. Assuming an annual drop-out rate of 20%, 114 patients per group will be required. The total required sample size would thus be n = 228. To allow possible subgroup analyses, a total of 300 patients will be recruited (n = 100 per center).

### Recruitment

Recruitment will be performed within the pediatric clinics of the participating centers. Both referred and in-house patients will be approached after routine examination was performed. Eligible patients and their parents will receive the study information and consent forms. Written consent will be given by the parents of the children. There will be a minimum time period of 48 hours between receipt of study information and consent.

### Allocation and blinding

If more than one primary molar is eligible, the decision as to which tooth will be included into the study will be performed using random number tables (WH) before treatment is commenced. Sequence generation will be performed after inclusion via random number tables (WH). Allocation will be performed via sealed opaque envelopes after the six-monthly recall examination (six months after selective excavation) by the treating dentists. Thus, both the first excavation step and the first re-examination will be performed blinded as to whether further excavation will be performed or not. Clinical follow-up examinations will also be performed blinded to treatment group allocation. Blinding during the second excavation step and blinding of patients as to which treatment was performed will not be possible, but patients will be informed not to reveal treatment allocation to the examiner during follow-up examinations.

### Outcomes

Outcomes will be assessed by the treating dentists or study nurses. The primary outcome of the study will be success (not requiring any re-intervention). Secondary outcomes will include survival (not requiring extraction), restoration integrity, assessed via modified USPHS-criteria [13], patients’ subjective assessment of the treatment using a visual analogue scale (for stepwise excavation, assessments will be performed twice and mean visual analogue scale results will be calculated), dentists’ and parents’ subjective assessment of the treatment using grades 1 (very good) to 6 (very bad) and initial and follow-up treatment costs (calculated by combining costs for staff, as assessed by time required for the procedures and the number of personnel involved, and costs for transportation of patients or materials).

### Intervention and data collection

In the first visit, a full assessment and intraoral examination will be performed. For patients who are possibly eligible, caries risk will be estimated [22] and dental anxiety assessed [23]. If informed consent is given, treatment will be provided in the second visit, with local anesthetics being applied according to individual needs. Removal of enamel and cavity preparation will be performed using water-cooled diamond instruments. Caries removal in the periphery including the enamel-dentinal junction will be performed using rose head burs and/or an excavator until hard, dry dentin remains. Pulpo-proximal caries will be removed until leathery, slightly moist and reasonably soft dentin remains. Operating dentists will be calibrated prior to study commencement regarding these criteria using extracted teeth. Moisture control will be performed using cotton rolls and continuous aspiration. Restoration will be performed adhesively with the use of a self-etching one-bottle adhesive (G-aenial bond, GC, Bad Homburg, Germany) and a compomer material (Dyract, Dentsply Detray, Konstanz, Germany).

**Figure 1:**
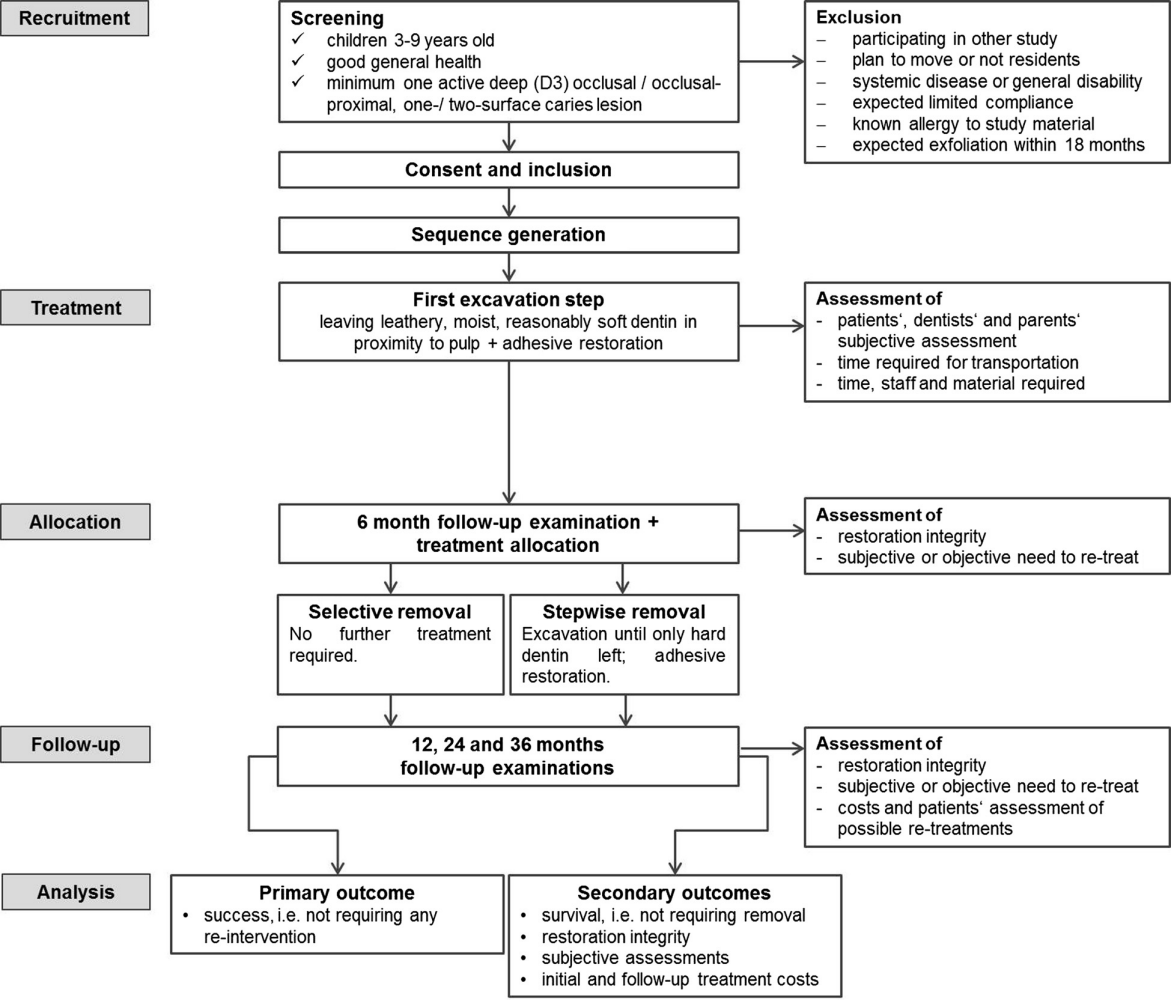
**Flowchart of the study.**

After six months, a follow-up examination will be performed. After the examination, allocation will be revealed. If allocated to stepwise excavation, the second excavation will be performed as described until only hard, dry dentin remains. Restoration will again be provided adhesively as described. All treatment steps will be documented photographically if possible (Figure 1).

If pulpal exposure occurs during excavation, a pulpotomy will be attempted. This will be provided according to dental routine, using ferric-sulfate for hemostasis and a calcium hydroxide dressing. Teeth with amputated pulps will be restored via stainless steel crowns if necessary. If no amputation is possible, teeth will be extracted. All retained teeth will be followed up on regardless of the treatment arm.

Data collection will be performed via case reports forms. The following data will be collected after the initial or second-step treatment:

1. Anesthetic and restorative material used,
2. Patients’ cooperation (1 to 4) [24],
3. Subjective assessment of patient (visual analogue scale),
4. Subjective assessment of parent (grade 1 to 6),
5. Subjective assessment of dentist (grade 1 to 6),
6. Transportation time and costs, and
7. Time, staff and material required for treatment.

During follow-up examinations, the following parameters will be assessed:

1. Tooth retreated at another clinic (yes or no),
2. Tooth exfoliated (yes or no),
3. Sensitivity and symptoms, and
4. Restoration integrity [22].

If retreatments are required, the cost of these will be assessed as well. Retained, retreated teeth will be followed up on to assess the survival of these teeth regardless of retreatment. A summary of performed procedures and recorded data can be found in Table 2.

**Table 2:** **Timing of measurements**

**Measures**	**Preoperative**	**First step**	**After six month/second step**	**Follow-up**	**Retreatment**
Demographics	x				
Dental history	x				
Caries risk	x				
ICDAS score	x				
Sensitivity testing	x	x	x	x	
Radiographic depth	x				
Radiographic endodontic status		(x)	(x)	(x)	(x)
Dental anxiety	x				
Cooperation		x	x		x
Subjective assessments		x	x		x
Costs (time, staff and material)		x	x		x
Clinical outcomes			x	x	x

### Data analysis and statistical evaluation

The statistical evaluation will be performed using SPSS 20.0. Descriptive statistics will be calculated according to distribution of data. Log rank tests will be used to compare success and survival between groups, with effects of possible confounders (age, gender, dental anxiety, cooperation, use of local anesthetics, caries risk, dental arch, first or second molar, surfaces and center) being assessed. Moreover, we will use two-sided independent t tests or Mann-Whitney U test (to compare visual analogue scale results and costs) as well as chi-square tests (to compare restorative integrity and subjective grading of treatments).

### Missing data

We anticipate various reasons for missing data (Table 3). Sample size calculation was performed accounting for possible loss to follow-up, that is, data missing at random. To prevent data missing not at random, case report forms have been designed in a way that enables and enforces complete reporting. Moreover, regular data control procedures will be performed for the early detection of missing data. Moreover, we will account for data not missing at random due to imbalanced loss to follow-up by handling drop-outs as non-success or non-survival using the intention-to-treat principle.

**Table 3:** **Handling of loss to follow-up**

**Level**	**Reason**	**Consequence**
Tooth	Complication	Complications leading to tooth loss will be counted as non-survival. Teeth with complications which allow the retention of the tooth will be count as non-success. Required follow-up treatments will be recorded and survived teeth will be followed up on.
	Lesion treated by other dentist	Tooth will be counted as non-success or non-survival.
	Tooth exfoliation	The last observation will be carried forward.
Patient	Adverse or serious adverse events related to treatment or material	Counteractive measures will be taken and events documented.
		Patient and tooth will be censored and the last observation carried forward.
	The patient cannot be examined (lost to follow-up)	Patient and survived tooth or teeth will be censored and the last observation carried forward.
	The patient decides to withdraw from the study.	Patient and tooth will be censored and the last observation carried forward.
Study	One to four patient(s) show adverse events related to treatment or material	Counteractive measures will be taken and events documented.
		Patient and tooth will be censored and the last observation carried forward. The study will be interrupted.
	More than four patients show adverse events or one patient shows a severe adverse event related to treatment or material	Counteractive measures will be taken and events documented.
		The study will be terminated. All patients and available teeth will be marked as censored and the last observation carried forward.

### Ethical considerations

#### Ethical approval

The protocol was approved by the ethical committee of the Charité - Universitätsmedizin Berlin, Germany, the Ernst-Moritz-Arndt University Greifswald (approval number: BB 112/14) and the RWTH University of Aachen (approval number: EK 283/14). In case of adverse and/or severe adverse events, the review board will be informed as outlined below. Similarly, the board will be contacted to approve significant protocol amendments. Both parents and patients will receive detailed verbal and written explanations regarding the study and the procedures therein involved. Informed written consent of the parents is required and should be given not earlier than 48 hours after study information.

#### Protocol amendments

All amendments to the protocol shall be agreed to by the principal clinical investigator and be recorded with a justification for the amendments. Amendments will be reviewed to determine the need for ethically reapproving the amended protocol. All deviations will be similarly reviewed. Changes and amendments will be recorded, including a detailed change history, and will eventually be reported.

#### Consent and assent

Patients and their parents will be informed about the study and will be requested to sign the informed consent forms not earlier than 48 hours after being provided with study information. Assent to participate will be documented in the case report forms.

#### Withdrawal

Patients and their parents will be informed that patients have the right to withdraw from the study at any time without giving reasons. Regardless of withdrawal, patients will be provided with any treatment in their best interest. Withdrawal will be documented and consent will be sought from participants to retain data collected up to the point of withdrawal.

### Dissemination of results

The results of this study will be published in international peer-reviewed journals. A summary of study results will also be saved at Clinicaltrials.gov to allow general access to obtained findings.

### Trial management

#### Oversight

Study initiation, implementation, evaluation and monitoring are supported by the Department of Operative and Preventive Dentistry, Charité - Universitätsmedizin, Berlin, Germany. Each participating center (Charité - Universitätsmedizin Berlin, RWTH Aachen and Ernst-Moritz-Arndt University Greifswald) will recruit and treat patients and document both treatments and follow-up assessments as outlined. For each center, local oversight by local investigators will be implemented.

#### Responsibilities

The principal investigator (FS) will oversee this study; act as main contact for all study communication and monitor trial procedures including documentation. Deviations from the protocol will be reported to him, who will be responsible for analyzing and approving or declining deviations. The local investigators of the two other centers (MP and RS) will be responsible for center-specific issues regarding the trial and constantly supervise the data collection, reporting and all active clinicians. We plan four trial group meetings: initiation and calibration, 12- and 24-monthly re-evaluations, 36-monthly/final evaluation.

#### Adverse events and code-breaking

The performed procedures, despite not being used by all dentists worldwide, can be regarded as clinical routine, especially for treating children [25,26]. We therefore expect adverse or severe advents to be similar to those associated with routine treatment. Specific study-related adverse or severe events will be allergy or severe reaction to a study material used (local anesthetics, bonding and/or compomer) or rare, uncommon reactions (unexpected bleeding, unexpected severe pain and/or uncontrollable anxiety) to or during the performed interventions. Any adverse event to treatments provided within the study will be investigated and reported to the ethics committee. In case of serious adverse events requiring un-blinding, code-breaking is possible. If the investigation is terminated prematurely or suspended, the principal clinical investigator (FS) will promptly inform the clinical investigators of all centers. Additionally, the ethics committee will also be informed immediately.

## Discussion

The treatment of deep caries lesions bears significant risks for the pulp of the tooth, including pulpal exposure, especially in deciduous teeth [1,10]. For the latter, two opposite strategies for treating deep caries are currently available. On the one hand, more conservative options aim at retaining pulpal integrity and health. On the other hand, more invasive strategies anticipate an eventual loss of pulpal vitality and perform pulpotomy instead. Whilst the latter treatment may be highly successful, requiring few re-interventions and often retaining primary teeth until their exfoliation [27], it might be both more burdensome for children and more costly compared with less invasive strategies. Moreover, it might not be feasible in many circumstances, requiring local or even general anesthetics and further, advanced restorations using, for example, preformed crowns. Given the polarized distribution of deep lesions both between and within countries [28], incomplete caries removal followed by direct restoration might be an option, which is applicable especially for conditions which, or patients who, do not allow for other options.

In the planned study, our main outcome variable will be success, followed by survival. Admittedly, both have to be seen as having limited precision and reliability: Since we omit regular radiographic evaluations and rely solely on clinically determined need to retreat, we might miss ‘failures’ and thereby systematically bias our results. However, our approach is both clinically relevant and ethically sound; in a clinical setting, regular radiographic re-examination is unlikely due to concerns of radiation. Moreover, the need for re-intervention would be determined subjectively too, especially when treating children, since only few dental practitioners would aim at endodontically treating asymptomatic teeth based only on a negative ‘vitality testing’ result [26,29,30].

In the planned study, retreated teeth will not be excluded from further analyses, but followed. Thus, we will be able to not only determine the initial efficacy of both treatments, but measure their long-term effects. These long-term effects have been found to determine both the retention time of the tooth and the total generated costs [2]. Moreover, they are additionally burdening children, and might have further effects regarding their attitudes toward the dentist.

In general, patients’ assessment of different therapies is relevant especially when treating children, since personal experiences or preferences shape both long-term attitudes and short-term compliance. The opinion of providers (dentists) might be relevant to implement less invasive caries treatments in general practice; only having the evidence (knowledge) is often insufficient for translating change into a primary care setting. Here, personal experience and attitudes are of high importance for the acceptance of a treatment [31-34]. Dentists’ attitudes regarding selective excavation have been additionally shaped by fear of restorative failure, especially if legal regulations such as guarantee times are in place [25]. We therefore aim at focusing not only on pulpal failures, but will also assess restoration integrity.

The mentioned regulative framework is put in place by political decision-makers. The latter are, again, only limitedly influenced by evidence and driven by several indirect factors like political reward, feasibility or justifiability of change [35,36]. Justifiability might be given if a new treatment option is more cost-effective than the established standard especially in a cost-driven environment with low overall priority like dental treatment [36]. Comparing the cost-effectiveness of selective versus stepwise excavation might further assist the implementation and clinical decision-making regarding treatment of deep caries lesions in deciduous molars.

## Trial status

The trial was registered at Clinicaltrials.gov and the study is open for recruitment.
